# Unleashing the potential of AI for pathology: challenges and recommendations

**DOI:** 10.1002/path.6168

**Published:** 2023-08-07

**Authors:** Amina Asif, Kashif Rajpoot, Simon Graham, David Snead, Fayyaz Minhas, Nasir Rajpoot

**Affiliations:** ^1^ Tissue Image Analytics Centre, Department of Computer Science University of Warwick Coventry UK; ^2^ School of Computer Science University of Birmingham Birmingham UK; ^3^ Histofy Ltd, Birmingham Business Park Birmingham UK; ^4^ Department of Pathology University Hospitals Coventry & Warwickshire NHS Trust Coventry UK; ^5^ Cancer Research Centre University of Warwick Coventry UK; ^6^ The Alan Turing Institute London UK

**Keywords:** artificial intelligence, computational pathology, histopathology, whole slide images, deep learning, machine learning

## Abstract

Computational pathology is currently witnessing a surge in the development of AI techniques, offering promise for achieving breakthroughs and significantly impacting the practices of pathology and oncology. These AI methods bring with them the potential to revolutionize diagnostic pipelines as well as treatment planning and overall patient care. Numerous peer‐reviewed studies reporting remarkable performance across diverse tasks serve as a testimony to the potential of AI in the field. However, widespread adoption of these methods in clinical and pre‐clinical settings still remains a challenge. In this review article, we present a detailed analysis of the major obstacles encountered during the development of effective models and their deployment in practice. We aim to provide readers with an overview of the latest developments, assist them with insights into identifying some specific challenges that may require resolution, and suggest recommendations and potential future research directions. © 2023 The Authors. *The Journal of Pathology* published by John Wiley & Sons Ltd on behalf of The Pathological Society of Great Britain and Ireland.

## The promise of AI in computational pathology

**Figure 1:**
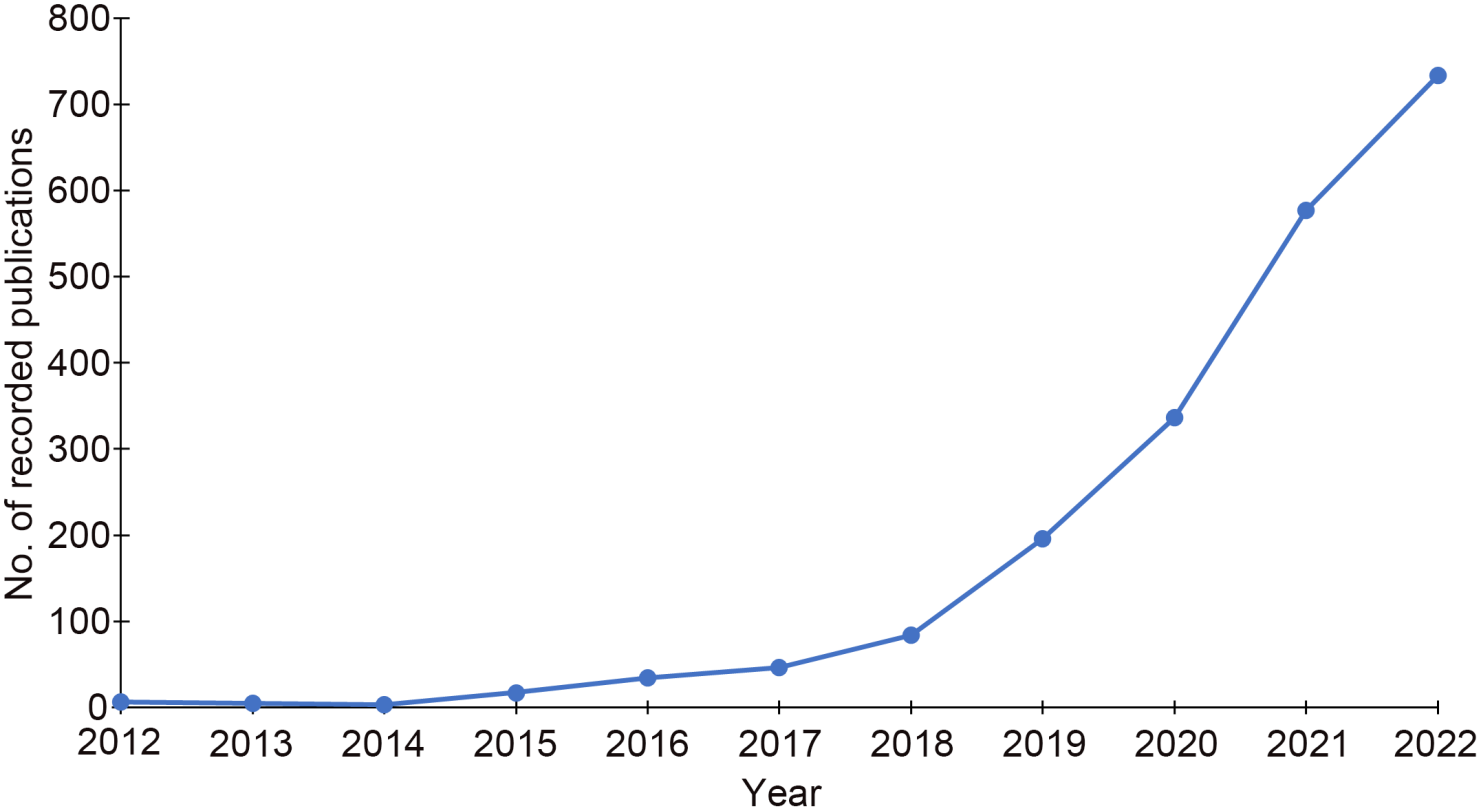
Number of research publications in AI‐based computational pathology recorded in PubMed over the last decade.

In recent years, there has been a notable surge of interest in the application of artificial intelligence (AI) for computational pathology (CPath) across various sectors including academia, industry, and healthcare. Research publications recorded on PubMed show more than a 100‐fold increase in AI‐based research activity in CPath during the period 2012–2022 (see Figure 1). The increase in literature and healthcare applications focused on AI‐powered computational pathology can be attributed to a variety of factors, such as the advancements in machine/deep learning (ML/DL) techniques, the digitization of tissue slides, the curation of large datasets, and the availability of high‐performance computing hardware. Typically, ML/DL methods for CPath are developed using tissue images with associated clinical metadata and/or annotations. These models hold the potential to assist medical professionals in making precise and efficient diagnoses as well as developing effective treatment plans for patients with cancer.

**Figure 2:**
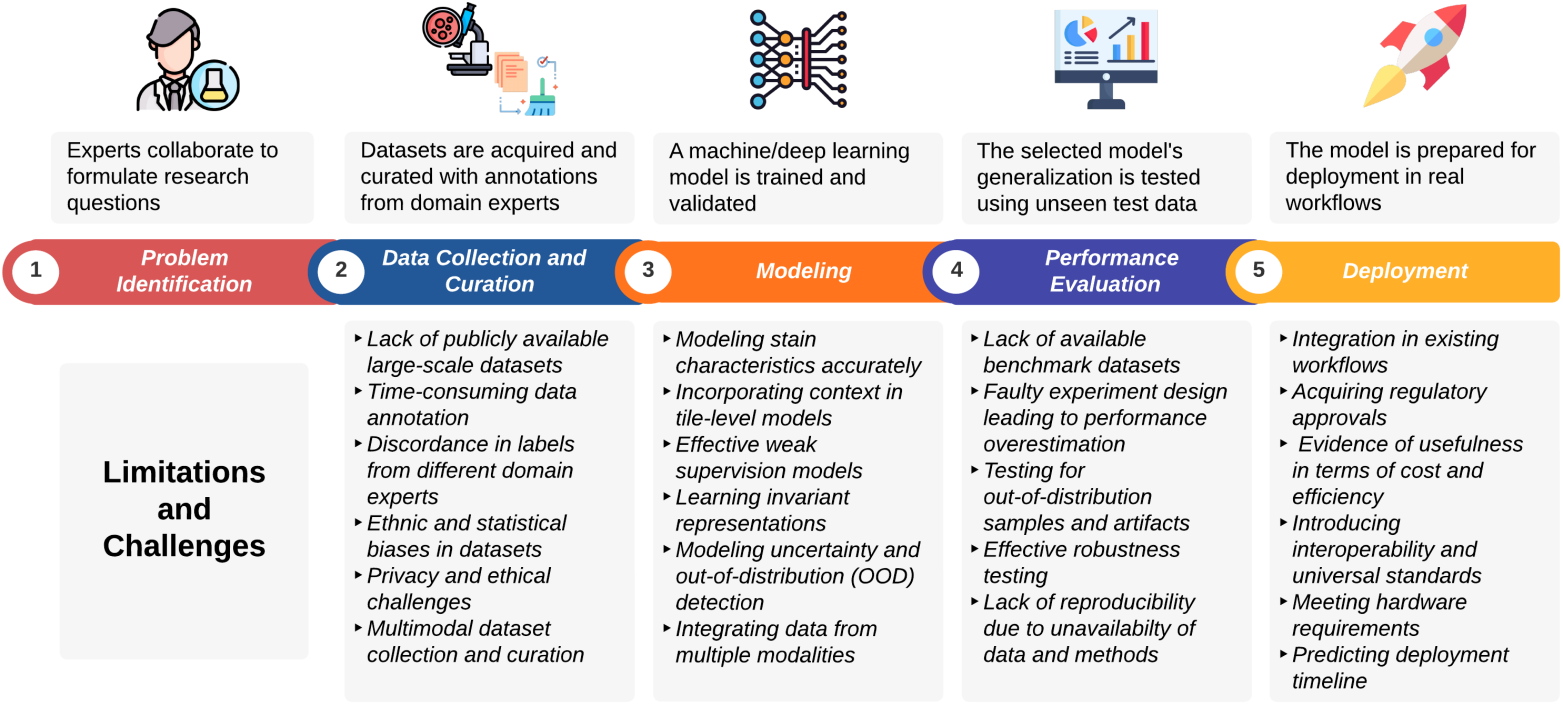
The conventional workflow followed in the development of a CPath system and challenges associated with each phase. The figure has been created using lucidchart.com. Publicly available icons from flaticon.com have been used in the figure.

Similar to other AI application areas, the conventional workflow for developing CPath methods consists of five stages (Figure 2). In the first stage, a research problem is formulated. This step typically involves active collaboration among domain experts (e.g. pathologists, oncologists, biomedical researchers) and data scientists. The second stage is curation of training, validation, and testing datasets to be used for model development and evaluation. In the third stage, a machine learning model is trained using the data, and the final model selection is conducted using the validation set. The model is kept blind to the test set in this stage to avoid performance overestimation. Once the model has been selected, its performance is evaluated on appropriate evaluation metrics using independent test set(s) in the fourth stage. The fifth and final stage is the deployment of the model in real‐world settings to assist the clinicians, consequently enhancing the existing diagnostic, prognostic, and treatment workflows.

**Figure 3:**
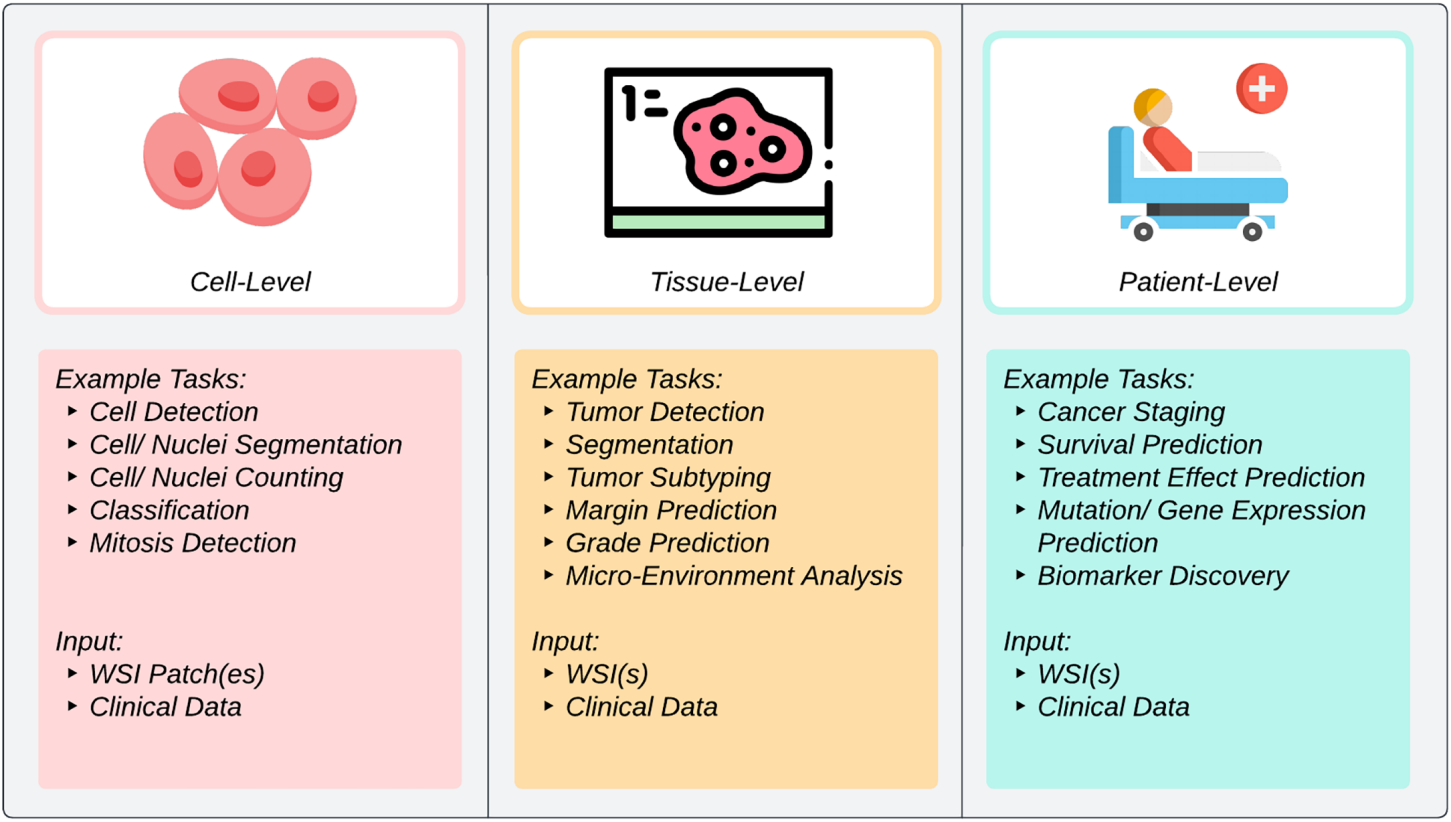
Categorization of CPath methods based on the level of analysis. The figure has been created using lucidchart.com. Publicly available icons from flaticon.com have been used in the figure.

In the past decade, AI has been used to model a wide spectrum of problems in histopathology, sometimes claiming *super*‐*human* performance [1, 2]. Several recent review articles have covered CPath research trends from various perspectives [3, 4, 5, 6, 7, 8, 9, 10, 11, 12, 13, 14, 15, 16, 17, 18]. Based on the level of analysis, application, and prediction variable(s), CPath algorithms can be broadly categorized into three groups: cell‐level, tissue‐level, and patient‐level (Figure 3). These algorithms are built using whole slide images (WSIs) along with corresponding clinical data. Processing a WSI as a whole is usually infeasible due to computational limitations, and therefore a commonly adopted approach for most methods in all three categories is to divide a WSI into smaller image patches or tiles before processing them. The cell‐level algorithms are designed to analyze individual cells and their features from WSIs or patches that have been extracted from WSIs. Examples include cell segmentation, detection, classification, and mitosis detection [19, 20, 21, 22, 23, 24, 25, 26, 27]. Such methods can assist pathologists in identifying any irregularities in the cellular landscape that might be indicative of the severity of disease and patient prognosis. Furthermore, the outputs of these algorithms can also be used in many downstream tasks such as tumor detection, cancer grading, and predicting patient outcomes. Tissue‐level CPath algorithms typically analyze entire regions of tissue in WSIs. The goal is to identify patterns and anomalies in different tissue regions that can be predictive of a disease or any relevant clinical variable. Examples of tissue‐level algorithms include detection and segmentation of different tissue types, cancer subtyping, and tumor margin prediction [28, 29, 30, 31]. Like cell‐level algorithms, the outputs of tissue‐level algorithms have been used in a number of downstream tasks such as tumor microenvironment analysis, cancer grade prediction, and patient survival analysis [32, 33, 34]. The patient‐level algorithms operate at the highest level of abstraction and usually utilize WSIs (one or more) and associated clinical and/or genomic annotations from a patient to generate predictions for diagnosis, prognosis, and suggesting appropriate treatment plans. Examples of such predictions include patient survival, treatment response, genetic expression/mutation, and the origin of primary tumor [35, 36, 37]. Regardless of the level of application, results of the analysis may need to be aggregated to generate higher‐level predictions [38].

The high accuracy figures reported in the literature across various application areas can be considered evidence of the immense potential of AI for successfully modeling CPath problems. The research community generally agrees on the potential of AI to revolutionize the field by enabling efficient and more accurate diagnoses and prognoses [39, 40]. Other major benefits of using CPath algorithms are their objectivity and reproducibility, in contrast to the subjective nature of a pathologist's visual examination, which can result in variations in interpretation among observers [41]. In addition, the significant investments made in the field through public and private funding contribute to the promise of AI technologies achieving breakthroughs in CPath, potentially leading to a considerable impact on the practices of pathology and oncology. The US Food and Drug Administration (FDA) has recently approved an AI‐based prostate cancer detection method with the potential to generate significant impact in the field [42].

While DL, both generally and for CPath, has gained enormous popularity, it is worth noting that the associated hype can lead to somewhat unrealistic expectations and potentially serious consequences if substandard technologies are adopted without proper scrutiny. To fully harness the true potential of AI in CPath, it is necessary to overcome several limitations in the current systems that are hindering their widespread clinical adoption [43]. For instance, a key challenge in the clinical uptake of CPath technologies is that they may not generalize well to new/unseen datasets, and therefore may not be ready for launch into the real‐world clinical settings [44]. Moreover, there are several other challenges in various phases of development, including, but not limited to, the scarcity of publicly available datasets and models, the absence of stringent and problem‐specific performance evaluation protocols, the lack of uniform standards and regulatory policies, and the reproducibility of methods, which could hinder the development of effective models for CPath. We discuss these and other associated challenges and possible solutions in detail in the following sections and conclude this review article with a list of open problems for future research in the field.

## Limitations, challenges, and recommendations

Similar to other application areas in AI/DL/ML, the typical lifecycle of a CPath project, after the problem has been formulated, can be divided into four major phases: data collection and curation, model development, performance evaluation, and deployment. In this section, we highlight the major limitations and challenges associated with each of these phases (see Figure 2). Overcoming these limitations and challenges is critical to integrating CPath systems effectively into both research and clinical workflows. To assist pathology readers, we present a glossary of AI‐related terminologies used in this review article in Table 1.

**Table 1:** Glossary of AI‐related terminologies used in this review article.

**Artificial intelligence (AI)**: The field concerned with developing computer systems that can perform tasks that require human intelligence
**Machine learning (ML)**: A subfield of AI that focuses on the development of techniques that enable machines to learn from data. It uses different algorithms to train models that can make predictions or take actions based on the patterns and insights found in the data
**Deep learning**: A subset of ML that focuses on the development and implementation of neural networks with multiple layers, called deep neural networks. Deep learning algorithms are designed to automatically learn hierarchical representations of data, extracting progressively more abstract features from raw input
**Supervised learning**: A paradigm of ML that uses labeled data for training models, meaning that the input data are paired with corresponding target labels. The algorithm learns to make predictions on unseen data based on the labeled examples
**Weakly supervised learning**: An ML paradigm that allows development of models from imprecise or inexact labels of training examples typically used when fine‐grained labeled data are limited, unavailable, or expensive to obtain. An example of this can be training a model to diagnose colorectal abnormalities with case‐level labels as opposed to using more precise regional or cell‐level annotations
**Generalization and overfitting**: The ability of a model to generate correct predictions on unseen data is called generalization. A model is said to have good generalization if it can correctly predict targets for real‐world test samples. A model is said to overfit on a dataset if it can generate correct predictions only for that dataset but fails otherwise
**Training**: The process of optimizing internal parameters of a machine learning model on training data so that it learns to produce desired outputs over these data points. Examples of internal parameters can be weights and biases of a neural network or decision cutoffs in a decision tree
**Validation**: The process of estimating the generalization performance of a machine learning model typically used for selecting optimal hyper‐parameters of a model for a given problem such as the number of neurons or the number of training cycles of a neural network. This is done by using performance metrics such as accuracy or area under the receiver operating characteristic curve over a validation set of examples that are not used in training directly
**Testing**: The process of estimating the real‐world predictive performance of a trained machine learning model on unseen data, i.e. data not used in training or validation
**Out‐of‐distribution (OOD)**: OOD refers to data or examples that differ significantly from the distribution of the training data that the model was exposed to during training. OOD data can be seen as samples that fall outside the scope of what the model has learned and may have limited or no representation in the training data. For example, if a model is trained using only colorectal biopsies, then samples from other tissues can be considered as out‐of‐distribution for this model

### Data

Collection and curation of datasets is the first and most important step in an ML study. In the absence of good quality datasets that accurately reflect the respective populations, it is very hard to develop effective models and conduct realistic performance assessment. We outline some of the challenges specific to CPath in terms of data collection, curation, and processing in the following subsections.

#### Large‐scale datasets – the need and challenges

CPath datasets typically consist of WSIs of tissue sections along with associated clinical and genomic data. WSIs are typically multi‐gigapixel in size, resulting in large storage and processing requirements [45]. However, despite the very large size of an individual WSI, WSI datasets often contain only a relatively small number of independent examples – i.e. these datasets are typically tall and thin [46]. Scarcity in terms of the number of independent examples combined with the problem of high dimensionality in CPath makes DL models highly prone to overfitting [47].

Given the *data‐hungry* nature of DL [48], large datasets are required for accurate modeling of problems, which is particularly challenging in CPath due to the need for specialized scanning equipment, quality checks, and trained technical staff and pathologists for labeling and annotation. This problem can be mitigated to some extent by learning low‐dimensional representations, but the need for sufficiently large datasets stands nonetheless [49, 50, 51, 52]. Initiatives such as The Cancer Genome Atlas (TCGA) have played a significant role in the development of CPath algorithms by providing publicly available WSIs along with associated genetic and clinical information [53]. Additional large‐scale publicly available datasets are required for developing effective DL solutions.

#### Data annotation and discordance

Precise cell‐level or region‐level annotations are crucial for training and evaluation in many supervised learning tasks in CPath [54]. However, acquiring such annotations can be expensive, tedious, and time‐consuming. Unlike natural images (i.e. non‐medical), where crowdsourcing‐like techniques can be used to acquire labels, trained personnel are required to annotate histopathology images accurately. Amgad *et al* [55] demonstrated that crowdsourcing from medical students for annotating cell nuclei in breast cancers was considerably accurate, hence providing a relatively more efficient framework for data annotation. However, the effectiveness of crowdsourcing for other types of annotations and more complex problems still remains elusive.

Interactive segmentation [22, 56] is an alternate approach to address this issue, as it can provide annotations for a wide range of objects of various scales and speed up the collection of annotations with minimal interaction from the expert annotator. Yet another approach is the synthetic generation of realistic high‐resolution tissue images [57], with associated annotations, with a realism score comparable to the pathologists.

Another related issue is the discordance among pathologist labels, due to the inherent subjectivity of visual assessment [58, 59, 60]. Discordant annotations, in addition to being a source of labeling noise, lead to a disagreement over what to be used as ground truth for supervised training and performance evaluation. Consensus results from multiple pathologists may offer a more reliable ground truth, but at an additional labeling cost [61].

#### Data biases

Biased data often lead to biased ML/DL models, which can have serious implications. For example, like other healthcare informatics data, CPath data are highly prone to ethnic bias [62, 63, 64, 65], referred to as the *health data poverty* problem [66]. Despite significant technological advancements, developing and underdeveloped parts of the world still lack sufficient infrastructure for generating digital histopathological and genomic data, leading to lack of representation from these regions. Population underrepresentation combined with the fact that histology and prevalence of cancers can vary highly across different races [67, 68, 69] not only raises concerns about universal generalization of models but also increases the gap in access and applicability of advanced tools and solutions to poorly represented ethnic groups. To overcome this issue, initiatives for data collection from underrepresented ethnicities are needed. Furthermore, CPath models need to be tested and corrected for potential racial biases [70].

CPath datasets, like other datasets, are prone to several statistical biases; for example, cancer survival datasets are vulnerable to biases such as immortal time bias, selection bias, and lead‐time bias [71, 72], all of which can lead to significant over‐/under‐estimation of treatment efficacy and the effects of other covariates of interest [73]. Most CPath survival studies lack analysis and correction for such biases, raising questions over the true effectiveness of the identified covariates. For such studies, adequately sized datasets corrected for such biases are needed to analyze the effects of covariates accurately. Several recommendations for addressing biases have been presented in [74].

#### Privacy and ethical challenges

Public concerns over the privacy of healthcare data may be regarded as the biggest cause of scarcity of publicly available datasets. Similarly, constraints around commercial use of the data may hinder deployment of advanced AI algorithms developed with such data. A related challenge is the implication of a participant's right to unenroll from a study at any time. Access revoking may require a patient's data to be removed not only from *all* related databases but also from *all* models that are trained using their data, i.e. making the models *unlearn* an example. There has been some work in developing unlearning techniques for models, but this is still an open problem in ML [75, 76, 77] with a need for effective and time‐saving solutions. AI in healthcare brings with it a number of ethical concerns, for instance, the potential to aggravate discrimination and inequality due to biased ML algorithms [78]. Another concern is that the *black box* nature of DL algorithms makes it difficult for the clinicians to trust and rely upon model predictions [79]. Comprehensive coverage of ethical challenges in incorporating ML models for healthcare and ethical issues in pathology is presented in [80, 81] and [82], respectively.

#### Multimodal data collection and curation

Recently, there has been a surge of interest in developing prognostic and predictive DL models for patients using WSIs. While these models have shown promise in predicting patient outcomes using WSIs, it is important to note that histopathology data alone may not provide a complete representation of a patient's expected survival, as histopathology data show only a partial view of a complex landscape. For effective modeling of such problems, additional information is needed, such as genomic and clinical data, thus highlighting the need for systems built on multimodal data. However, collecting, curating, and collating multimodal datasets is far from straightforward. In addition to challenges associated with collaboration, correspondence, and data sharing among different centers for creating such datasets, another issue is that not all types of data may be available for all patients. This can lead to a significant number of missing entries in a dataset, necessitating the development of specialized modeling techniques with support for heterogeneous and missing data for downstream analysis. Published research exploring the integration of multimodal data, such as [83, 84], has shown promising results.

### Modeling

The goal of AI/ML/DL models in CPath is to learn a suitable representation of tissue morphology and architecture associated with disease group/phenotype, molecular genotype, treatment effects, other omics signatures, and important objects (e.g. cell nuclei, micro‐vessels, tubules) in a tissue slide. In this section, we discuss challenges specific to modeling in CPath.

#### Modeling stain characteristics

Many existing approaches fail to model the domain‐specific characteristics of images in CPath and treat them as *natural RGB* images. Such approaches do not explicitly model the fact that WSIs are obtained through a multi‐step process that has a significant impact on their characteristics. Variations in tissue processing steps such as chemical fixation or freezing, dehydration, embedding, and staining can change the visual characteristics of the tissue slide in a non‐uniform and non‐linear manner across tissue types and laboratories well before the tissue slide is scanned to produce WSIs. *Stain variation* is typically handled *post hoc* by stain estimation, normalization, or augmentation approaches to generate RGB images. Although stain augmentation can be effective when there are sufficient data, there is a need for methods that explicitly capture the characteristics of stain absorption and the associated non‐linearities across tissues. Such methods are necessary to develop models that are invariant to these factors and demonstrate improved generalization capabilities [85, 86].

#### Context and multi‐resolution nature of WSIs

Pathologists typically analyze histological patterns at various magnification levels for visual assessment, taking into account the contextual information to aid in their decision‐making. Due to their sheer size, a WSI is often divided into image tiles (or *patches*) at a specific magnification, making the problem of modeling context in WSIs more challenging compared with images from other domains. Training and inference are both typically performed with limited context captured by individual patches, with the underlying assumption being that each patch is an *independent* data point. In addition, CPath algorithms also face the well‐known *signal‐frequency uncertainty* dilemma: the broader the context, the less precise the localization of a region or object. A multi‐resolution approach can integrate predictive information at multiple levels, at the cost of an increase in model complexity – potentially requiring more training data for effective learning. Another compromise is a distributed attention mechanism that can integrate information across multiple spatial locations and magnification levels. Existing methods in computational pathology have attempted to address these challenges to some degree [87, 88]. However, to the best of our knowledge, no existing method has demonstrated its ability to model context effectively across a variety of computational pathology tasks.

#### The case for weak or no supervision

The size of WSIs poses a major problem in the form of computational bottlenecks in performing gradient computations while training DL models. Several existing CPath methods employ patch‐level analysis, which assumes that the patch labels are available and can provide a direct supervisory signal for effective training. However, obtaining patch‐level labels can be very time‐consuming and typically only WSI‐level labels are available for training, making a compelling case for the use of *weak supervision techniques*. Weakly supervised CPath algorithms [50, 89, 90, 91, 92, 93, 94, 95] aggregate patch‐level prediction scores by different mechanisms, such as majority voting, average pooling, or multiple instance learning. The success of these approaches depends on the nature of the ML task and the validity of assumptions underlying these approaches. Recently, self‐supervised learning methods [96, 97, 98, 99] that exploit supervisory signals in the data itself with the help of domain‐specific as well as domain‐agnostic tasks have proven to be successful for effective tumor detection with limited available annotations. However, development of truly generalizable weakly supervised or self‐supervised approaches remains an open problem [28].

#### Learning invariant representations

AI methods in CPath require an effective representation of input images that is robust to variations resulting from factors such as rotation, translation, slide preparation, staining, and scanner characteristics, in order to allow the model to generalize well to unseen test data [86]. The invariances can be learned through various augmentation strategies, self‐supervised learning [96, 97], and contrastive learning [100]. In addition to the symmetries associated with classical images such as translation and rotation, CPath models also need to cater for domain‐specific invariances, including invariances associated with *technical* changes such as stain and scanner characteristics as well as histological properties underlying a prediction task [101]. For example, variations in breast tissue density or fat content across population types can impact tumor subtype classification models. Such variations, if not factored in the development of CPath models, can lead to generalization failure. Although several approaches have modeled technical invariances, explicitly modeling histological variations in CPath models and learning domain‐specific invariant representations need to be explored further.

#### Modeling uncertainty and out‐of‐distribution (OOD) detection

Modeling label uncertainties in model training and generating uncertainty (or confidence) scores with inference are key requirements for the practical utility of CPath models. This can be achieved by calibrating model predictions or by developing methods that can generate confidence scores associated with each prediction. Confidence scores can enable predictive models to ‘*know what they don't know*’, detect OOD test examples, and abstain from generating a decision in such cases [102, 103]. A few existing approaches have addressed this issue [104, 105, 106, 107]. However, this dimension of CPath model development requires further attention for their use in practice.

#### Multimodal data integration

Development of models that utilize multimodal data from heterogeneous sources such as radiology, pathology images, genetic sequencing and transcriptomics, multi‐spectral and multiplexed imaging, spatial transcriptomics, clinical data, clinical letters, and laboratory reports is an open area of research in computational diagnostics. Mining such data can reveal interesting associations and lead to the discovery of novel biomarkers and early diagnosis of multiple diseases. Some approaches have been proposed for the fusion of patho‐radiomic and patho‐genomic features [83, 108, 109]. However, in order to model such solutions as ML problems, a key challenge is the availability of linked multimodal datasets. As a consequence, approaches such as learning using privileged information that assume that data from some modalities may only be available during training, but not during inference, can be very helpful. Development of such models requires close interaction between national and international health providers and ML researchers. One solution may be to provide an anonymized public data exchange that can accelerate the development of such solutions.

### Performance evaluation

AI models in CPath with their promise of enhanced efficiency and accuracy herald the dawn of an era for data‐driven AI for the practice of cellular pathology in clinical and pharmaceutical workflows. Their deployment in practice, however, requires stringent performance evaluation as the decisions produced by these models are expected to have implications on patients’ health and drug discovery roadmaps. In conventional settings, researchers attempt to estimate a model's accuracy on unseen data by using cross‐validation protocols and testing on independent sets [110]. However, models may still not generalize well to unseen data [111], often due to lack of robust performance evaluation [112]. Below, we cover some of the limitations and challenges concerning realistic performance evaluation and rigorous validation of CPath models.

#### Lack of available benchmark datasets

One of the biggest hurdles in accurate performance assessment of CPath models is the shortage of openly available, high‐quality, and broadly representative benchmark datasets [113], leaving researchers with no choice but to evaluate their models over data that might be pragmatically available but may not be a full representation of the real world. For fair evaluation and comparison of methods, benchmark datasets should capture characteristics of real test data ‘*from the wild*’ with a sufficient number of examples following the expected test data distribution and ideally representing all segments of the population. A benchmark dataset should also follow the FAIR principle of data management [114], i.e. it should be findable, accessible, interoperable, and reusable. Excellent pathology‐specific recommendations for curating high‐quality test sets have been discussed in [115].

#### Experimental design

While conducting performance evaluation of a model, the most important part is to ensure that experimental design is appropriate for realistic and reliable performance evaluation. For example, in the context of currently popular complex and multi‐stage DL pipelines, a significant number of studies lack fair baseline comparisons and ablation studies justifying the need for added complexity. Furthermore, while performing a comparison among different methods for solving a problem, it should be ensured that the experimental conditions are consistent for all methods [115]. This includes using the same data examples for training and inference, the same level of hyperparameter optimization, and not fixing splits that favor one method over the other.

A common technique used in ML/DL is to keep on tuning the model until an acceptable or ‘*superior’* performance is achieved on the test set. Such practice can lead to false discovery due to multiple testing instead of good generalization. To prevent this, it is recommended to follow an approach similar to the one used in grand challenges, where the test set is used only once, so that the test set is not used indirectly for model selection as this can potentially result in overfitting on the test data. Overfitting on the test data leads to an overestimation of the model's true predictive performance. Therefore, reuse of test sets should be discouraged. If the method does not perform well and re‐tuning of parameters is performed, additional unseen data should be used in testing. This, however, can be challenging due to data scarcity as discussed above.

There is no fixed rule for dataset division into training and validation sets. The optimal splits can vary based on factors such as dataset size, diversity, problem complexity, etc. The conventional practice is to find a split that provides an adequate number of data points for training while ensuring a suitable number and quality of data points for proper validation.

Another factor that can cause overestimation of performance results in CPath models is the patient‐level overlap in training and test samples. Extending the argument further, *broad validation* consisting of unseen test data from external centers should be preferred to *narrow validation*, where unseen data from the same center can be used for testing purposes [116].

#### OOD and sanity tests

Digitized WSIs of tissue slides often require cleaning up and removing of irrelevant and noisy regions such as pen markings, background, and other artifacts. In practice, CPath models can encounter WSIs with artifacts as well as out‐of‐distribution (OOD) WSIs [117]. The model should be able to distinguish OOD samples from noisy images and images of interest. There exists limited research on developing models that can *abstain* from prediction for data samples that are either too noisy or do not belong to the distribution of interest.

#### Robustness analysis

DL models have been shown to be vulnerable to adversarial attacks and small perturbations in the input [118, 119, 120]. Even highly accurate models may lack robustness towards small variations and therefore fail miserably. Though adversarial attacks are less likely for healthcare models, small perturbations are quite probable due to variations in factors such as staining, scanning environments, and equipment [121, 122, 123]. Therefore, cross‐validation and independent set testing, though necessary, may not be entirely sufficient for performance assessment. *Fragility analysis* to evaluate how a model would respond to changes is also required in CPath and other healthcare applications. A model should be deemed deployable only if it demonstrates adequate robustness to adversarial attacks and small perturbations in inputs.

#### Reproducibility and repeatability

Several scientific domains [124, 125, 126, 127], including ML in general and its application to healthcare in particular, are facing a major reproducibility crisis. There are a large number of methods with SOTA (state of the art) accuracies being reported frequently in the literature, with a significant fraction that cannot be reproduced or repeated because of several factors. Two major causes of the lack of reproducibility in CPath are unavailability of data and models, often citing privacy concerns or due to commercial conflicts, and missing or incomplete preprocessing details. In particular, for CPath, this includes information regarding data preparation, quality check measures for WSIs, discarded examples/cases, stain normalization techniques, patch extraction and selection, etc. To ensure successful reproducibility, details such as model initialization techniques, data augmentation, batch sizes, hyperparameters and data splits are needed. Not mentioning these details can lead to issues in successful replication of results. To handle issues with reproducibility and repeatability in CPath, recommendations in [128] inspired from the FAIR principles [114] can be followed.

### Deployment

The ultimate aim of a CPath algorithm is to automate and assist with the pathologist's assessment of tissue slides. Additionally, CPath methods can also be employed for deep mining and discovery of novel histological patterns for prognostic and predictive biomarkers. Either way, in order to ensure that CPath algorithms are deployed in real‐world clinical and pharmaceutical workflows, the following aspects of deployment must be considered.

#### Workflow integration

CPath solutions should ideally be integrated into the existing clinical and pharmaceutical workflows in order to automate or assist the pathological decision‐making processes. Careful integration with existing laboratory information management (LIM), electronic health record (EHR), image management (IM) systems, and/or trial databases may appear to be a low‐tech problem but is crucial for seamless workflow in routine pathology and oncology practice. Often, the launch platform must be clinically validated and have regulatory approvals too. Launching a separate CPath application, a common paradigm followed by several current CPath solution providers, that runs side‐by‐side all the above systems can only be the second‐best option.

#### Reimbursement model

The reimbursement for CPath solutions is not currently available in most countries [15, 18]. This is a major barrier to CPath adoption and deployment in those countries, as it means that laboratories and hospitals cannot recoup the costs of implementing CPath solutions. The adoption of CPath solutions requires that such solutions are financially incentivized to sustain their uptake. There is a need for evidence to demonstrate the value of CPath [129], in order for payers to develop reimbursement policies and procedures that reflect these benefits. The Digital Pathology Association's reimbursement task force is working with payers and various stakeholders to develop a fair reimbursement model.

#### Validation and regulatory approvals

A CPath solution that can be deployed in routine clinical practice needs to have been validated rigorously to generate clinical evidence required for confidence of and buy‐in from clinicians in the solution. Most healthcare systems require the solution to comply to ISO standards and pass regulatory approvals, such as the Food and Drug Administration (FDA) in the United States and In Vitro Diagnostics (IVD) in the European Union. Going forward, as CPath algorithms become more autonomous, we may need stringent regulatory approvals considering the question of responsibility in cases where the autonomous algorithms fail [11]. This need is further exacerbated by the aforementioned challenges associated with reliability and robustness.

#### Evidence for usefulness

Before a practical CPath solution can be deployed in practice, there should be sufficient and robust evidence for its usefulness in terms of efficiency gains, higher accuracy, and cost savings. Typically, well‐designed health economic studies are required to generate evidence for efficiency gains and cost savings. Lack of such evidence may hamper the wider buy‐in from the user community and may also make it difficult for the laboratory or hospital management to justify investment in deployment of the solution, given the relatively high initial setup cost of the digital and computational pathology infrastructure.

#### Generalizability and interoperability

There is some evidence to suggest that DL algorithms do not perform equally well on images from different scanners or even different versions of the same scanners. CPath solutions must develop and demonstrate interoperability for various types of WSI formats generated by different slide scanners in order to help ensure that they are able to deal with this particular source of variation that is known to result in *domain shift* and are not biased towards or against pixel data from one or more image formats. Standardization of output formats for decisions made and annotations done by CPath algorithms (e.g. in GeoJSON format) will further enable interoperability of algorithms and aid with workflow integration. It is hoped that international industry–academic–clinical cooperative efforts for finalization of interoperability standards (such as the WSI DICOM standard) will help to address these challenges.

#### Computational infrastructure and resource requirements

CPath models are generally computationally expensive due to the relatively large size of WSIs. In this context, at least the following three models have recently emerged: (1) the ***cloud***‐based data‐to‐compute model, whereby WSIs are typically shipped to and processed in the cloud; this model offers the attractive feature of *pay‐per‐use* options without requiring significant compute‐heavy investment but may give rise to potential data sharing and privacy concerns; (2) the ***central*** compute‐to‐data model, whereby data are shipped into a central repository and various compute solutions are brought over to be executed within the repository environment; this model is attractive for central repositories and for users without access to compute and storage resources but is likely to incur high initial setup cost; and (3) the ***federated*** learning model, whereby the DL model is trained locally without having to share the data, a global model is put together by merging the local models, and then the global model is shared with all the contributing sites; a slight caveat of this model is that it requires sufficiently powerful computing resources at all contributing sites to be able to train local models.

There is no doubt that AI offers the potential to address the increasingly serious issue of pathologist shortage in most countries, especially low‐to‐middle‐income countries (LMICs). Recent work has also shown that scanners can be miniaturized and images from mobile phones can be used for point‐of‐care diagnostics in low‐resource settings [89, 130]. We hope that further technological advances in AI model optimization, storage, and networking may lead to reduced hardware requirements and address data sharing concerns.

Understanding the environmental impact of AI infrastructure usage has an increasingly important role as there is a pressing need to develop solutions that rely on sustainable practices. We need infrastructure usage and model development practices that enable efficient use of large datasets, model reuse, and data‐efficient and parameter‐efficient AI methods that have low energy consumption.

#### Deployment timeline and the spectrum of mundanity

**Figure 4:**
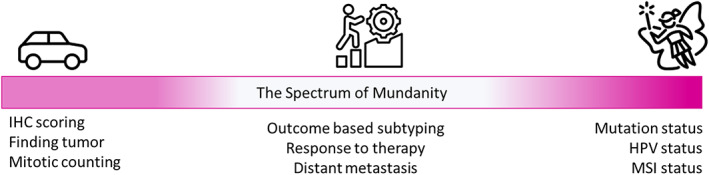
Complexity of CPath tasks in terms of their mundanity. Publicly available icons from flaticon.com and icons8.com have been used in the figure.

A question that is frequently asked is: which AI applications are likely to become practical and widely available in the near future? To answer this question, we would like to refer the reader to Figure 4, which we term as the *spectrum of mundanity*. At one end of the spectrum, we have challenges such as identifying tumors in a biopsy or lymph node, and counting the number of mitotic cells in pathology samples. These are objective problems that pathologists can typically solve with a high degree of accuracy and reproducibility. At the opposite end of the spectrum, there are relatively obscure tasks that pathologists are unable to solve with anything more than a subjective ‘*gut feeling*’, for example, predicting the molecular status based solely on visual examination of a histology slide, potentially reducing the need for slow and expensive molecular testing especially useful in low‐resourced settings. In the middle of the spectrum, there are relatively difficult tasks that often reflect complex interplay between tumor and host, which may be difficult for humans to observe in a reproducible manner. These tasks require large amounts of data for algorithm development and must undergo prospective large‐scale multi‐centric validation with long follow‐up periods. Examples of such tasks include risk scoring for malignant transformation, local recurrence, or distant metastasis of cancer, as well as predicting a patient's response to a particular therapy. We postulate that CPath solutions for tasks on the two ends of the spectrum that match (left) or surpass (right) the pathologist performance are the ones that will be deployed in routine practice sooner than those in the middle.

## Conclusions and future directions

The emerging field of CPath holds significant promise in enabling the discovery of known histological patterns, as well as uncovering previously unknown cellular and tissue architectural motifs. This breakthrough technology has the potential to revolutionize cellular pathology‐based diagnostics, prognostics, treatment selection, and patient stratification, with significant implications for patient care. There have been various positive developments in DL‐based CPath in recent years, showing great promise for facilitating enhancement in pathological assessment of tissue slides. However, some challenges remain to be addressed to make the vast majority of CPath methods truly generalizable and applicable in practice.

To conclude this article, we list some research directions and open questions as follows:

1. **Causality and mechanistic insights**: Although existing CPath models can predict mutation status from an image, they do not inform whether morphological features associated with the prediction are indeed a result of the mutation or not. We conjecture that causal modeling with an ability to estimate individual treatment effects will lead to a mechanistic understanding of predictive image‐based CPath biomarkers.
2. **Interpretability and explainability**: While the lack of interpretability and explainability is a common challenge for the DL domain, it has a more pronounced impact in pathology since the decision making can influence diagnosis, prognosis, treatment planning, and drug discovery roadmaps. More research is needed towards incorporation of interpretability and explainability in CPath models.
3. **Standards and guidelines**: We believe that closer collaboration and engagement between clinical, academic, industrial, and patient/public stakeholders is a pressing need of the hour. In particular, such a collaboration will lead to the development of standards and guidelines for (1) storing, archiving, reading, collection, curation, and sharing of WSIs with linked image‐level and patient‐level (e.g. clinical and genomic) annotations; (2) robust validation and generalizability of CPath models; and (3) deployment, readouts, and interpretability of AI algorithms.
4. **Linking disparate data modalities and data sharing via secure platforms**: We believe that building appropriate connectors between different data hosting platforms (PACS, EHR, etc.) for different data modalities is the key to linking multimodal datasets. Once datasets have been linked and curated, they could be accessed for AI algorithm development and validation via a trusted research environment (TRE) or secure data environment (SDE) if sharing of the data is not an option.
5. **AI use guidelines and legal responsibility**: The rapid progress of AI in cellular pathology during the last decade is in sharp contrast to the lack of clear guidelines and use cases (e.g. the Royal College of Pathologists datasets) on how to make use of the AI solutions in cellular pathology. There are also concerns among some quarters that a new breed of pathologists using AI algorithms may gradually become so reliant on algorithms that they may lose their ability to recognize some nuanced histological patterns that they may have picked otherwise. Although it is a bit too early to remark on the likelihood of this eventuality, this is a predictable consequence of technology and one that will need to be addressed through CPD training and quality assurance. To benefit from the promise of CPath, there is a need to produce AI use guidelines that incentivize pathologists to benefit from technology while avoiding overreliance on AI [131]. There is also a clear need to develop legal responsibility policies and guidelines, to address the current regulatory gap [132], owing to the rapidly emerging CPath solutions. There is an essential requirement to understand the moral and legal responsibilities of AI‐based decisions [133] as CPath solutions could potentially disrupt traditional practice where decisions may depend on AI.
6. **Outcome‐based subtyping**: Histological subtypes for various types of cancers are often based on a combination of morphological and architectural patterns, signifying the different types and the degree of malignancy. The disciplines of pathology and oncology, and consequently the cancer patients, stand to benefit from steering the focus of CPath‐based histological subtyping towards outcome‐based subtyping. Shifting the focus away from matching the existing histological subtyping also offers an opportunity to explore the extraction of subvisual insights from the data via a *latent* representation which may not be apparently perceivable.
7. **New imaging modalities and time to thaw**: Recently developed imaging modalities, such as spatial transcriptomics and MUSE as well as volumetric 3D tissue imaging, offer promise for future research avenues in CPath. However, as mentioned earlier, sufficiently sized multi‐centric repositories need to be set up for effective modeling especially when using DL. Development of effective ML models using frozen tissue sections is challenging due to generally poorer image quality, which complicates the detection of tissue and cellular morphological patterns. Despite the clinical significance of these approaches, development of ML models for frozen tissue images remains relatively unexplored.
8. **The real test and the Turing test**: Finally, the CPath community should perhaps consider organizing high‐quality challenge contests on larger problems with a focus on multimodal data analysis, federated data analysis, generalizability, OOD detection, learning with abstinence, robustness analysis, and artificial general intelligence (AGI) solutions. Systematic concordance and discordance studies (similar to the IBM Watson for Oncology [134]) are lacking that compare clinical decision making against the algorithm's decision making and not just for individual sub‐tasks (e.g. segmentation). We propose work on a Turing test for pathology, similar to the one proposed for cancer [83], whose objective will be to observe how AI solutions can assist in decision making for diagnosis, prognosis, and treatment planning. We realize that the design of such a test will be a long process, but initially the test can be designed for an individual task (e.g. cancer detection, cancer grading, and TILs grading) and later on can be evolved for the ability to handle a group of tasks along the lines of AGI, as discussed above.

The future of CPath is promising, but its success depends on the community's ability to bridge the gap between the estimated performance of CPath models and their actual performance in real‐world applications. This will be a critical step towards successful deployment of CPath into real‐world clinical and pharmaceutical workflows, as well as ensuring its long‐term sustainability.

## Author contributions statement

AA, KR, FM and NR conceptualized the manuscript. NR was responsible for the overall supervision of the writeup. All authors contributed to the drafting, writing, reviewing and editing of the manuscript.
